# Covid-19 and Increased Risk of Physician Suicide: A Call to Detoxify the U.S. Medical System

**DOI:** 10.3389/fpsyt.2022.791752

**Published:** 2022-02-09

**Authors:** Sophia E. Kakarala, Holly G. Prigerson

**Affiliations:** ^1^Cornell Center for Research on End-of-Life Care, Department of Medicine, Weill Cornell Medicine, New York, NY, United States; ^2^Department of Medicine, Weill Cornell Medicine, New York, NY, United States

**Keywords:** physician suicide, COVID-19, physician burnout, Post-Traumatic Stress Disorder, medical licensing, physician mental health

## Abstract

Suicide among physicians is a longstanding problem, with risk factors exacerbated by the coronavirus disease 2019 (COVID-19) pandemic. In this article, we explore suicidal thoughts and behaviors among physicians and risk factors created or intensified by the work environment, such as overwork and loss of autonomy. We discuss the ways in which the COVID-19 pandemic has made the medical work environment more stressful (e.g. greater exposure to traumatic experiences and employment insecurity) and, consequently, elevated physician suicide risk. We also review evidence that the medical system in the United States has not adequately protected physicians' mental health. Lack of confidentiality, stigma, cost, and time, as well as intrusive medical licensing applications, remain barriers to physicians seeking help. Work pressures imposed by insurance companies and financial incentives to increase revenue while cutting costs compound physicians' work stress. We conclude that system-wide changes to the practice of medicine and policies regarding healthcare delivery are needed to improve physicians' work environments, as is research addressing the impact of the interventions to reduce their suicidal risk. The proposed changes, and greater access to timely and confidential mental health services amid and in the aftermath of the pandemic, may prove promising approaches to reduce physicians' suicide risk.

## Introduction

The longstanding problem of physician suicide has drawn renewed attention during and in the wake of the COVID-19 pandemic. Popular belief has long held that physicians die by suicide more often than the general population, at a rate up to twice as high (1). An analysis of violent deaths in the United States (U.S.) between 2012 and 2016 contradicts this (2), but is consistent with prior studies which find that work stressors are more often linked to suicide in physicians (3). There is, thus, both opportunity and urgent need to address aspects of the work environment that influence physician suicide risk. Reform is particularly pressing in the wake of a pandemic that has seen physicians and other medical workers exposed to greater levels of danger, including provision of care to patients who have not yet received COVID-19 vaccines (4), upheaval (5), and immense suffering (6), as well as, in some cases, workplace closures and job instability, which itself poses elevate suicide risk. The American system of privatized medicine has made its hospitals and practices uniquely vulnerable to the economic vicissitudes of the pandemic (7). In this article, we discuss how the work environment in the U.S. and the COVID-19 pandemic together have posed alarming threats to the mental health and increase the suicide risk of practicing physicians.

This article will focus on the suicide risk factors experienced by physicians in the U.S., who, according to one 2019 meta-analysis, were more than 1.3 times as likely than physicians anywhere else in the world to kill themselves (8). It is our aim to note the factors that exacerbate suicide risk among physicians and point to potentially promising ways to reduce this risk. Physician mental health is a pressing concern globally (8), and we by no means wish to suggest that the U.S. is exceptional in the pressures placed on medical workers. However, many risk factors and barriers to care are modifiable only on a country-by-country basis. We focus attention on the salient, and possibly modifiable, suicide risks to physicians posed by their workplace environment and exacerbated by the corporatization of healthcare (9). We argue that systemic reform is needed to prioritize physicians' mental health and, thereby, reduce their risk of suicide.

## Systemic Risk Factors Among U.S. Physicians

The prevalence of job-related stress among physicians who die by suicide accords with robust research showing that a medical career can create or exacerbate mental health problems. While certain personality traits, such as maladaptive perfectionism, are associated with physicians developing mental health problems (10), the conditions of medical training appear to cultivate psychological distress among many physicians. An erosion of trainee's mental health begins within months of starting medical school (11). The often extreme deprivations and stresses of medical training, including emotional abuse (12) and unhealthy long shifts (13), erode mental health and inculcate self-blame, which heighten suicide risk (14).

Graduating medical students enter an increasingly corporatized work environment (9). Simultaneously, mental ill-health linked, or potentially linked, to work is on the rise among physicians. One international meta-analysis found that self-reported depression among medical residents increased between 2006 and 2015, with between 20.9 and 43.2% experiencing depression or depressive symptoms (15). Burnout, an occupational problem comprising exhaustion, cynicism, detachment and feelings of ineffectiveness (16), also rose among U.S. physicians between 2011 and 2014 (17). The problem of physician burnout is global, with physicians worldwide working in increasingly unforgiving conditions (18). Though some studies find it is not directly linked to suicide (19), burnout has symptomatic and conceptual overlaps with depression (2, 20), and is associated with medical error (19), itself a physician suicide risk factor (21).

In particular, burnout rates are elevated among physicians in emergency, family, and general internal medicine (17) – specialties in which physicians are seeing increasing patient volume due to misaligned financial incentives (22). Dissatisfaction with work-life balance among U.S. physicians is also increasing (17). Documentation burden, which has grown with Medicare, insurance, and hospital reporting requirements, is also a strong predictor of burnout (23). According to one study of psychiatry residents, documentation burden was a greater factor in burnout than clinical rotation, sleep, or other lifestyle factors (24). Further, declining professional autonomy (9), often a byproduct of healthcare consolidation in the U.S. (25), undermines a sense of control over one's own schedule and practice environment, which has been found to be the single strongest predictor of physicians' job satisfaction (26). A study of physicians working in the U.K.'s National Health System supported the connection between lack of autonomy and high levels of burnout, with emotional exhaustion and depersonalization mediating the relationships between autonomy and psychological symptoms (27). It is concerning, therefore, that in recent decades, U.S. physicians have experienced a loss of autonomy, combined with increasing time pressure and a comparatively heavy clerical burden (28, 29).

Physicians' increasing exposure to risk factors for burnout and other work-related syndromes is disconcerting in light of the need to reduce suicide risk. According to Joiner's interpersonal theory of suicidality, a suicide attempt results from three aspects: lack of belonging, perceived burdensomeness, and the acquired capability for suicide (30). These align with the primary aspects of burnout, including depersonalization (resulting in withdrawal) and a reduced sense of personal accomplishment (16), which may well precede social alienation and perceived burdensomeness. (31) validated the link between work dissatisfaction and feelings of ineffectiveness and physician suicidality, as mediated by anhedonia. These conditions may be exacerbated by modern trends in healthcare, such as the lack of autonomy discussed above (9) and the increased use of impersonal quality metrics (32) to evaluate physicians' performance.

## Mental Health Risks Posed by the Pandemic

Existing suicide risk factors for physicians are compounded by the COVID-19 pandemic. A 2015 study of suicidality in physicians found that experiencing traumatic medical events predicted the acquired capability for suicide (33), supporting the theory that physicians' exposure to pain and horror meets one of Joiner's pre-conditions for suicide (30, 34). The effect may be especially stark for physicians who are not accustomed to treating critically ill patients, such as thosethose redeployed to critical care settings from non-acute specialties during the COVID-19 pandemic. In addition, exposure to dangerous working conditions and the emotional impact of providing futile care (35) may fuel feelings of cynicism and detachment, possible precursors to the thwarted belonging and self-perceived inefficacy that complete the triad of pre-conditions posing risk for suicide (30). Indeed, one international study found that depressive symptoms were elevated among healthcare workers who were redeployed to the ICU (36). Other proven psychiatric risk factors during the pandemic include inadequate personal protective equipment (PPE), which was shown to be associated with both depression and anxiety (36, 37); and being a healthcare worker in the U.S. or U.K., which was also associated with depression (36). Symptoms of Post-Traumatic Stress Disorder (PTSD) were common among healthcare workers who treated COVID-19 patients in China and Italy (38, 39), and a survey at one New York City institution in April 2020 classified 57% of staff as suffering acute stress (40), a prodrome to PTSD, which itself is a risk factor for suicide (41).

Early data have not shown an increase in overall suicide deaths due to the COVID-19 pandemic (42). However, data are not yet available on suicide rates for physicians, some of whom have worked in crisis conditions since the beginning of the pandemic and continue to do so with emergence of new COVID-19 variants (43). Moreover, both the mental health consequences and practical changes wrought by the pandemic will persist. For example, medical workers who treated SARS patients during a 2003 outbreak in Beijing reported elevated depressive symptoms up to 3 years later (44). In practical terms, COVID-19 intensified financial pressure on U.S. rural and independent hospitals and practices (45) and accelerated the vertical consolidation of healthcare practices by corporations (46). The employment-related fallout of COVID-19 was evident in a September 2020 poll in which 30% of 2,334 physicians surveyed reported feeling hopeless due to the pandemic's effects on their employment (47) – an effect specific to the vulnerabilities of privatized medicine.

## Barriers to Access and Attempts to Overcome Them

The stigma of mental illness in the medical profession (48) is reinforced by medical license, job, and malpractice insurance applications (49), which may scrutinize a physician's entire psychiatric history. Physicians who report a past or present mental illness risk being subjected to monitoring of their behavior or barred from practicing altogether (50). The effect of invasive applications is profound. Half of physician respondents to one survey believed that they had met criteria for a mental illness but had not sought help; 44% of those cited fear of reporting to a licensing or hospital board (50). Despite increased attention to physician mental health during the pandemic, a national poll of emergency physicians in October of 45 found that 57% would fear for their jobs if they sought mental healthcare, and 73% agreed there was stigma around doing so (51). Physicians are forced into circuitous tactics, such as seeking treatment in a different city, self-prescribing, or paying cash to avoid insurance claim records (50). These tactics may be prohibitively expensive for medical students and residents (52). Finally, and significantly, scheduling constraints may make it difficult to get timely or regular treatment (53). Barriers to help-seeking combine with tragic results: physicians who die by suicide are half as likely as suicide victims in the general population to have been receiving mental health care prior to their deaths (3).

Suicide prevention programs at medical schools and hospitals have tried, with varying success, to address privacy, time, and cost barriers to therapy. Proven approaches include recruiting outside providers to provide free or discounted therapy sessions without filing insurance claims (54) and storing users' records outside of the hospital's electronic health record (55). The Interactive Screening Program (ISP), designed by the American Foundation for Suicide Prevention, encrypts user's identities to allow them to correspond anonymously with counselors (56). A program at the University of California, San Diego's medical school based on the ISP has proven successful at identifying and referring at-risk persons (57). However, response and referral uptake from large-scale screening can be low (57), highlighting the need for multiple avenues to treatment. Significantly, although time to pursue and receive help has been found to be the greatest barrier to physician help-seeking (53), we did not find a single hospital that has provided protected time for physicians to access mental health care.

## Future Research Directions

While privatized medicine is frequently discussed in terms of its impact on medical spending (58) and patient access (59), rarely is it examined with relation to physician mental health or suicide risk. There is a need for research to compare physician suicide risk in the U.S. with that in other healthcare systems to identify ways that these systems affect the mental health of medical care providers. For example, physician burnout rates may be more than three times higher in the U.S. than in Europe (18). While many papers recommend organizational fixes to reduce burnout by improving the working environment (60), before-and-after studies of such organizations are rare.

Further, despite a long history of interest in physician suicide in the medical literature, data are still lacking and some subgroups of U.S. physicians who face unique challenges to their mental wellbeing remain overlooked. As one example, female physicians have a relatively high suicide rate (61), but little is known about why this gender disparity exists. Future research is needed to elucidate the roots of sex differences in the rates of physician suicide. The fact that physicians more often complete suicide when they attempt it (62) suggests a need for safeguards against access to lethal means such as fire-arms and self-prescribed medications. In addition, non-white physicians report experiencing racism from patients and colleagues (63). These work conditions may undermine feelings of self-worth and pose risks that should be explored in future research. And, because physicians who are immigrants from another country experience unique stressors-including a visa process that restricts their employment options, xenophobia, and having their professional qualifications questioned (63), immigration status should be included in studies on physician mental health. Future research should focus on the ways in which physicians' work environment affects their mental health in general and their risk of taking their lives, more specifically.

Workplace circumstances that undermine autonomy and control over one's work conditions and restrict personal time to care for parents, children, or spouses; long shifts; substantial administrative or clerical load; and parental burden (64) may heighten physician suicide risk. The COVID-19 pandemic further highlighted the importance of working conditions and workplace culture to healthcare workers' wellbeing. Many healthcare workers reported both inadequate PPE and threats of retribution for calling out unsafe working conditions (65); the aftereffects of these conditions on job satisfaction and feelings of belonging should be examined. Healthcare workers may also have been troubled by the feeling that they were providing futile care, which is associated with burnout (35) and evocative of the helplessness symptomatic of PTSD (66). Given that healthcare workers who were redeployed to the ICU during COVID-19 and who perceived their training as inadequate have been found to be at higher risk of depression (36), the relationships between secondary traumatization, self-perceived medical errors, moral distress (67) and suicide risk factors should be examined. In addition, examining job-related suicide risk factors among physicians whose practices were financially damaged during COVID-19 and those in workplaces that were consolidated is warranted. Such studies could make use of the Beck Depression Inventory to measure perceived burdensomeness and thwarted belonging among a wide sample of physicians (31) to determine the mediating and/or moderating role of burdensomeness and belonging on physician suicide risk. Given high rates of burnout and quitting among medical workers (68), studies should make a special effort to include those who retired or changed jobs after experiencing the pandemic and explore their reasons for leaving the workforce.

## Discussion

Research on physician suicide is hampered by the impossibility of knowing the events that led up to any one death, and the danger of tritely attributing suicides to a specific cause. Nevertheless, some facts are clear: COVID-19 has intensified a wide range of stressors for medical workers; physicians' mental health problems go undertreated; and systemic changes to the medical workplace over the past few decades are adversely affecting physicians' mental health. Female physicians (61), those who work in rural areas (51), and those in under-resourced practices or hospitals (45) may be at particularly elevated risk.

Employment circumstances that create stress and burnout, which already contribute to physicians' suicide risk (69), must be addressed to prevent their lethal combination with the stressors of the pandemic. Physician wellbeing experts recommend the use of clinician float pools to allow adequate time-off, supporting the careers of part-time physicians, and including physician wellbeing in institutional metrics (70). Mental health screening or counseling programs must eliminate privacy concerns and cost barriers (55). Changes to working conditions should reduce documentation requirements (71), lengthen the time spent with patients (72); bolster physicians' independence (26, 70, 73, 74) and reduce overscheduling, which may be the greatest barrier to accessing mental health care (53).

Such measures, on the surface, run contrary to the profit motives that drive healthcare (9). Hospitals and other employers tend to lean instead on generic “wellness” programs that target individual health behaviors without concomitant organizational change (75, 76). Given this pattern – and the shift of many physicians' roles to employee (9), intensified by mergers and acquisitions in the wake of the pandemic (46)-physicians might benefit from joining unions (77, 78), which are shown to improve employees' health and control over working conditions (79). Membership in a union or physicians' advocacy organization may also counteract feelings of isolation or alienation, one of the conditions of suicidality according to Joiner's theory (30). The legislation needed to ban licensing applications from including questions that deter help-seeking (80, 81) may be attainable only through collective self-advocacy. So too may restorative changes to physicians' working lives.

Occupational health research frequently presents physician mental illness as a detriment to profit or productivity (82, 83). Solutions focused on treatment or wellness put the onus on workers to adapt themselves to the changing demands of the workplace. We suggest that the U.S. healthcare system should adapt its model for the practice of medicine so that it prioritizes and supports physician's wellbeing. A return to the pre-pandemic status quo, in which mental illness risk factors for physicians were high (73), could be catastrophic. COVID-19 has laid bare a longstanding problem: the U.S. medical system undermines physicians' needs (84) while restricting their autonomy and options for self-help. It is tragic that those who spend years training to care for others are so often themselves neglected when it comes to their mental health. In the turmoil resulting from the pandemic, there exists a rare opportunity to raise awareness of, advocate for, and implement policies that promote physicians' occupational and mental health. Doing so may be the best medicine to reduce the risk of physician suicide.

## Data Availability Statement

The original contributions presented in the study are included in the article/supplementary material, further inquiries can be directed to the corresponding author/s.

## Author Contributions

SK researched and wrote the manuscript. HP conceived of the manuscript, edited it, and contributed writing. Both authors contributed to the article and approved the submitted version.

## Funding

This work was supported by grants from the National Cancer Institute (CA197730; HP) and the National Institute of Mental Health (MH121886; HP).

## Conflict of Interest

The authors declare that the research was conducted in the absence of any commercial or financial relationships that could be construed as a potential conflict of interest.

## Publisher's Note

All claims expressed in this article are solely those of the authors and do not necessarily represent those of their affiliated organizations, or those of the publisher, the editors and the reviewers. Any product that may be evaluated in this article, or claim that may be made by its manufacturer, is not guaranteed or endorsed by the publisher.

## References

[1] Anderson P. Doctors' Suicide Rate Highest Of Any Profession. WebMD. Available online at: https://www.webmd.com/mental-health/news/20180508/doctors-suicide-rate-highest-of-any-profession (accessed May 2018).

[2] Ye GY Davidson JE Kim K Zisook S. Physician death by suicide in the United States: 2012–2016. J Psychiatr Res. (2021) 134:158–65. 10.1016/j.jpsychires.2020.12.064 33385634

[3] Gold KJ Sen A Schwenk TL. Details on suicide among US physicians: data from the national violent death reporting system. Gen Hosp Psychiatry. (2013) 35:45–9. 10.1016/j.genhosppsych.2012.08.005 23123101 PMC3549025

[4] Edwards E. “We Are Getting Clobbered”: Six Months into COVID-19, Doctors Fear What Comes Next. NBC News (2020). Available online at: https://www.nbcnews.com/health/health-news/we-are-getting-clobbered-six-months-covid-19-doctors-fear-n1232410 (accessed June 30, 2020).

[5] Grimm CA. Hospital Experiences Responding to the COVID-19 Pandemic: Results of a National Pulse Survey, March 23-27, 2020 (OEI-06-20-00300) (2020).

[6] Palazzolo J DeRosa A. The Coronavirus Crisis In Doctors' Own Words: “A Flood Of Death I Cannot Manage.” The Wall Street Journal. Available online at: https://www.wsj.com/articles/a-flood-of-death-that-i-cannot-manage-the-coronavirus-crisis-in-doctors-own-word*s-11586272173* (accessed May, 2020).

[7] Rubin R. COVID-19's crushing effects on medical practices, some of which might not survive. JAMA. (2020) 324:321–3. 10.1001/jama.2020.11254 32556122

[8] Dutheil F Aubert C Pereira B Dambrun M Moustafa F Mermillod M. Suicide among physicians and health-care workers: a systematic review and meta-analysis. PLoS ONE. (2019) 14:e0226361. 10.1371/journal.pone.0226361 31830138 PMC6907772

[9] Salmon JW Thompson SL. The Corporatization of American Healthcare: the rise of corporate hegemony and the loss of professional autonomy. Springer Nature. (2021). 10.1007/978-3-030-60667-1

[10] Thomas M Bigatti S. Perfectionism, impostor phenomenon, and mental health in medicine: a literature review. Int J Med Educ. (2020) 11:201–13. 10.5116/ijme.5f54.c8f8 32996466 PMC7882132

[11] Kalmoe MC Chapman MB Gold JA Giedinghagen AM. Physician suicide: a call to action. Mo Med. (2019) 116:211–6. 31527944 PMC6690303

[12] Dyrbye LN Thomas MR Shanafelt TD. Medical student distress: causes, consequences, and proposed solutions. Mayo Clin Proceed. (2005) 80:1613–22. 10.4065/80.12.1613 16342655

[13] Temple J. Resident duty hours around the globe: where are we now?. BMC Med Educ. (2014) 14:S8. 10.1186/1472-6920-14-S1-S8 25559277 PMC4304287

[14] Keilp JG Grunebaum MF Gorlyn M LeBlanc S Burke AK Galfalvy H. Suicidal ideation and the subjective aspects of depression. J Affect Disord. (2012) 140:75–81. 10.1016/j.jad.2012.01.045 22406338 PMC3375058

[15] Mata DA Ramos MA Bansal N Khan R Guille C Di Angelantonio E. Prevalence of depression and depressive symptoms among resident physicians: a systematic review and meta-analysis. JAMA. (2015) 314:2373–83. 10.1001/jama.2015.15845 26647259 PMC4866499

[16] Maslach C Leiter MP. Understanding the burnout experience: recent research and its implications for psychiatry. World Psychiatry. (2016) 15:103–11. 10.1002/wps.20311 27265691 PMC4911781

[17] Shanafelt TD Hasan O Dyrbye LN Sinsky C Satele D Sloan J. Changes in Burnout and Satisfaction With Work-Life Balance in Physicians and the General US Working Population Between 2011 and 2014. Mayo Clinic proceedings. (2015) 90:1600–13. 10.1016/j.mayocp.2015.08.023 26653297

[18] Kumar S. Burnout and doctors: prevalence, prevention and intervention. Healthcare. (2016) 4:37. 10.3390/healthcare4030037 27417625 PMC5041038

[19] Menon NK Shanafelt TD Sinsky CA Linzer M Carlasare L Brady K. Association of physician burnout with suicidal ideation and medical errors. JAMA. (2020) 3:e2028780. 10.1001/jamanetworkopen.2020.28780 33295977 PMC7726631

[20] Bianchi R Verkuilen J Schonfeld IS Hakanen JJ Jansson-Fröjmark M Manzano-García G. Is burnout a depressive condition? a 14-sample meta-analytic and bifactor analytic study. Clin Psychol Sci. (2021) 9:579–97. 10.1177/2167702620979597

[21] Goldman ML Shah RN Bernstein CA. Depression and suicide among physician trainees: recommendations for a national response. JAMA Psychiatry. (2015) 72:411–2. 10.1001/jamapsychiatry.2014.3050 25738529

[22] Kelen GD Wolfe R D'Onofrio G Mills AM Diercks D Stern S. Emergency department crowding: the canary in the health care system. NEJM Catalyst. (2021). Available online at: https://catalyst.nejm.org/doi/full/10.1056/CAT.21.0217

[23] Melnick ER Dyrbye LN Sinsky CA Trockel M West CP Nedelec L. The association between perceived electronic health record usability and professional burnout among us physicians. Mayo Clin Proceed. (2020) 95:476–87. 10.1016/j.mayocp.2019.09.024 31735343

[24] Domaney NM Torous J Greenberg WE. Exploring the association between electronic health record use and burnout among psychiatry residents and faculty: a pilot survey study. Acad Psychiatry.(2018) 42:648–52. 10.1007/s40596-018-0939-x 29785625

[25] Jauhar, S,. Shouldn't Doctors Control Hospital Care? The New York Times. Available online at: https://www.nytimes.com/2017/10/10/opinion/shouldnt-doctors-control-hospital-care.html (accessed May 26, 2021).

[26] Freeborn DK. Satisfaction, commitment, and psychological well-being among HMO physicians. West J Med. (2001) 174:13–8. 10.1136/ewjm.174.1.13 11154654 PMC1071220

[27] Khan A Teoh KR Islam S Hassard J. Psychosocial work characteristics, burnout, psychological morbidity symptoms and early retirement intentions: a cross-sectional study of NHS consultants in the UK. BMJ Open. (2018) 8:e018720. 10.1136/bmjopen-2017-018720 30037857 PMC6059335

[28] Fred HL Scheid MS. Physician burnout: causes. consequences, and (?) cures. Tex Heart Inst J. (2018) 45:198–202. 10.14503/THIJ-18-6842 30374225 PMC6183652

[29] Holmgren AJ Downing NL Bates DW Shanafelt TD Milstein A Sharp CD. Assessment of electronic health record use between US and Non-US health systems. JAMA Intern Med. (2021) 181:251–9. 10.1001/jamainternmed.2020.7071 33315048 PMC7737152

[30] Joiner TE Van Orden KA Witte TK Selby EA Ribeiro JD Lewis R. Main predictions of the interpersonal-psychological theory of suicidal behavior: empirical tests in two samples of young adults. J Abnorm Psychol. (2009) 118:634–46. 10.1037/a0016500 19685959 PMC2846517

[31] Loas G Lefebvre G Rotsaert M Englert Y. Relationships between anhedonia, suicidal ideation and suicide attempts in a large sample of physicians. PLoS ONE. (2018) 13:e0193619. 10.1371/journal.pone.0193619 29584785 PMC5870971

[32] Ofri D. Quality measures and the individual physician. N Engl J Med. (2010) 363:606–7. 10.1056/NEJMp1006298 20818853

[33] Fink-Miller EL. An Examination of the Interpersonal Psychological Theory of Suicidal Behavior in Physicians.*Suicide Life Threat Behav*. (2015) 45:488–94. pmid:25530088 10.1111/sltb.12147 25530088

[34] Cornette MM deRoon-Cassini TA Fosco GM Holloway RL Clark DC Joiner TE. Application of an interpersonal-psychological model of suicidal behavior to physicians and medical trainees. Arch Suicide Res. (2009) 13:1–14. 10.1080/13811110802571801 19123105

[35] Lambden JP Chamberlin P Kozlov E Lief L Berlin DA Pelissier LA. Association of perceived futile or potentially inappropriate care with burnout and thoughts of quitting among health-care providers. Am J Hosp Palliat Care. (2019) 36:200–6. 10.1177/1049909118792517 30079753 PMC6363893

[36] Khajuria A Tomaszewski W Liu Z Chen J-H Mehdian R Fleming S. Workplace factors associated with mental health of healthcare workers during the COVID-19 pandemic: an international cross-sectional study. BMC Health Serv Res. (2021) 21:262. 10.1186/s12913-021-06279-6 33743674 PMC7981382

[37] Shanafelt T Ripp J Trockel M. Understanding and addressing sources of anxiety among health care professionals during the COVID-19 pandemic. JAMA. (2020) 323:2133–4. 10.1001/jama.2020.5893 32259193

[38] Di Tella M Romeo A Benfante A Castelli L. Mental health of healthcare workers during the COVID-19 pandemic in Italy. J Eval Clin Pract. (2020) 26:1583–7. 10.1111/jep.13444 32710481

[39] Lin K Yang BX Luo D Liu Q Ma S Huang R. The mental health effects of COVID-19 on health care providers in China. Am J Psychiatry. (2020) 177:635–6. 10.1176/appi.ajp.2020.20040374 32605443

[40] Shechter A Diaz F Moise N Anstey DE Ye S Agarwal S. Psychological distress, coping behaviors, and preferences for support among New York healthcare workers during the COVID-19 pandemic. Gen Hosp Psychiatry. (2020) 66:1–8. 10.1016/j.genhosppsych.2020.06.007 32590254 PMC7297159

[41] Krysinska K Lester D. Post-traumatic stress disorder and suicide risk: a systematic review. Arch Suicide Res. (2010) 14:1–23. 10.1080/13811110903478997 20112140

[42] Sinyor M Knipe D Borges G Ueda M Pirkis J Phillips MR. Suicide risk and prevention during the covid-19 pandemic: one year on. Arch Suicide Res. (2021) 1–6. 10.1080/13811118.2021.1955784 34425066

[43] Kennedy K Marcelo M. ‘There Are Only So Many Beds’: COVID-19 Surge Hits Hospitals. The Associated Press. Available online at: https://apnews.com/article/joe-biden-health-florida-coronavirus-pandemic-38917e4fd073c8142df15de2d8102a24 (accessed August 5, 2021).

[44] Liu X Kakade M Fuller CJ Fan B Fang Y Kong J. Depression after exposure to stressful events: lessons learned from the severe acute respiratory syndrome epidemic. Compr Psychiatry. (2012) 53:15–23. 10.1016/j.comppsych.2011.02.003 21489421 PMC3176950

[45] Khullar D Bond AM Schpero WL. COVID-19 and the financial health of US hospitals. JAMA. (2020) 323:2127–8. 10.1001/jama.2020.6269 32364565

[46] Gustafsson L Blumenthal D. The Pandemic Will Fuel Consolidation in U.S. Healthcare. Harvard Business Review. Available online at: https://hbr.org/2021/03/the-pandemic-will-fuel-consolidation-in-u-s-health-care (accessed March 21, 2021).

[47] The Physicians Foundation. 2020 Survey of America's Physicians: COVID-19 Impact Edition. Available online at: https://physiciansfoundation.org/research-insights/2020physiciansurvey (accessed September 18, 2020).

[48] Mehta SS Edwards ML. Suffering in silence: mental health stigma and physicians' licensing fears. Am J Psychiatry Resident. (2018) 13:2–4. 10.1176/appi.ajp-rj.2018.1311018723190

[49] Gold KJ Shih ER Goldman EB Schwenk TL. Do US Medical Licensing Applications Treat Mental and Physical Illness Equivalently?. Family Med. (2017) 49:464–7. 28633174

[50] Gold KJ Andrew LB Goldman EB Schwenk TL. “I would never want to have a mental health diagnosis on my record”: A survey of female physicians on mental health diagnosis, treatment, and reporting. Gen Hosp Psychiatry. (2016) 43:51–7. 10.1016/j.genhosppsych.2016.09.004 27796258

[51] American College of Emergency Physicians. Mental Health Among Emergency Physicians. Available online at: https://www.emergencyphysicians.org/globalassets/emphysicians/all-pdfs/acep20_mental-health-poll-analysis.pdf (accessed May 26, 2021).

[52] Grischkan J George BP Chaiyachati K Friedman AB Dorsey ER Asch DA. Distribution of medical education debt by specialty, 2010–2016. JAMA Intern Med. (2017) 177:1532–5. 10.1001/jamainternmed.2017.4023 28873133 PMC5820693

[53] Rangel EL Castillo-Angeles M Kisat M Kamine TH Askari R. Lack of routine healthcare among resident physicians in New England. J Am Coll Surg. (2020) 230:885-92. 10.1016/j.jamcollsurg.2019.11.005 31765695

[54] Thompson D Goebert D Takeshita J. A program for reducing depressive symptoms and suicidal ideation in medical students. Acad Med. (2010) 85:1635–9. 10.1097/ACM.0b013e3181f0b49c 20881686

[55] Ey S Moffit M Kinzie JM Brunett PH. Feasibility of a comprehensive wellness and suicide prevention program: a decade of caring for physicians in training and practice. J Grad Med Educ. (2016) 8:747–53. 10.4300/JGME-D-16-00034.1 28018541 PMC5180531

[56] Mortali M Moutier C. Facilitating help-seeking behavior among medical trainees and physicians using the interactive screening program. J Med Regul. (2018) 104:27–36. 10.30770/2572-1852-104.2.27

[57] Zisook S Young I Doran N Downs N Hadley A Kirby B. Suicidal ideation among students and physicians at a U.S. medical school: a healer education. assessment and referral (HEAR) program report. OMEGA J Death Dying. (2016) 74:35–61. 10.1177/0030222815598045

[58] Whaley CM Zhao X Richards M Damberg CL. Higher medicare spending on imaging and lab services after primary care physician group vertical integration. Health Aff. (2021) 40:702–9. 10.1377/hlthaff.2020.01006 33939518 PMC9924392

[59] Kanter GP Segal AG Groeneveld PW. Income disparities in access to critical care services. Health Aff. (2020) 39:1362–7. 10.1377/hlthaff.2020.00581 32744946

[60] Montgomery A Panagopoulou E Esmail A Richards T Maslach C. Burnout in healthcare: the case for organisational change. BMJ. (2019) 366:l4774. 10.1136/bmj.l4774 31362957

[61] Schernhammer ES Colditz GA. Suicide rates among physicians: a quantitative and gender assessment (meta-analysis). Am J Psychiatry. (2004) 161:2295–302. 10.1176/appi.ajp.161.12.2295 15569903

[62] Arnetz BB Hörte LG Hedberg A Theorell T Allander E Malker H. Suicide patterns among physicians related to other academics as well as to the general population. results from a national long-term prospective study and a retrospective study. Acta psychiatr Scand. (1987) 75:139–43. 10.1111/j.1600-0447.1987.tb02765.x 3494382

[63] Serafini K Coyer C Brown Speights J Donovan D Guh J Washington J. Racism as experienced by physicians of color in the health care setting. Fam Med. (2020) 52:282–7. 10.22454/FamMed.2020.384384 32267524

[64] Stewart DE Ahmad F Cheung AM Bergman B Dell DL. Women physicians and stress. J Womens Health Gend Based Med. (2000) 9:185–90. 10.1089/152460900318687 10746522

[65] Sengupta S. With Virus Surge, Dermatologists And Orthopedists Are Drafted For The E.R. The New York Times. Available online at: https://www.nytimes.com/2020/04/03/nyregion/new-york-coronavirus-doctors.html (accessed April 3, 2020).

[66] American Psychiatric Association. Diagnostic and Statistical Manual of Mental Disorders (4th ed., text revision). The Association (2000).

[67] Williams RD Brundage JA Williams EB. Moral injury in times of COVID-19. J Health Serv Psychol. (2020) 2:1–5. 10.1007/s42843-020-00011-4 32363349 PMC7195905

[68] Yong, E,. Why Health-Care Workers Are Quitting In Droves. The Atlantic. Available online at: https://www.theatlantic.com/health/archive/2021/11/the-mass-exodus-of-americas-health-care-workers/620713/ (accessed November 16, 2021).

[69] Stehman CR Testo Z Gershaw RS Kellogg AR. Burnout, drop out, suicide: physician loss in emergency medicine, part I. West J Emerg Med. (2019) 20:485–94. 10.5811/westjem.2019.4.40970 31123550 PMC6526882

[70] Linzer M Levine R Meltzer D Poplau S Warde C West CP. 10 bold steps to prevent burnout in general internal medicine. J Gen Intern Med. (2014) 29:18–20. 10.1007/s11606-013-2597-8 24002633 PMC3889939

[71] West CP Dyrbye LN Shanafelt TD. Physician burnout: contributors, consequences and solutions. J Intern Med. (2018) 283:516–29. 10.1111/joim.12752 29505159

[72] Rothenberger DA. Physician burnout and well-being: a systematic review and framework for action. Dis Colon Rectum. (2017) 60:567–76. 10.1097/DCR.0000000000000844 28481850

[73] Ariely D Lanier WL. Disturbing trends in physician burnout and satisfaction with work-life balance: dealing with malady among the nation's healers. Mayo Clin Proceed. (2015) 90:1593–6. 10.1016/j.mayocp.2015.10.004 26653295

[74] Lyon C English AF Chabot Smith P. A team-based care model that improves job satisfaction. Fam Pract Manag. (2018) 25:6–11. 29537246

[75] Schrijver I Brady KJ Trockel M. An exploration of key issues and potential solutions that impact physician wellbeing and professional fulfillment at an academic center. PeerJ. (2016) 4:e1783. 10.7717/peerj.1783 26989621 PMC4793321

[76] Rider EA Gilligan MC Osterberg LG Litzelman DK Plews-Ogan M Weil AB. Healthcare at the crossroads: the need to shape an organizational culture of humanistic teaching and practice. J Gen Intern Med. (2018) 33:1092–9. 10.1007/s11606-018-4470-2 29740787 PMC6025655

[77] Carlson R. Is there a physician union in your future? Fam Pract Manag. (1999) 6:21–5. 10344929

[78] Scheiber N. Doctors Unionize To Resist The Medical Machine. The New York Times. Available online at: https://www.nytimes.com/2016/01/10/business/doctors-unionize-to-resist-the-medical-machine.html (accessed January 9, 2016).

[79] Hagedorn J Paras CA Greenwich H Hagopian A. The role of labor unions in creating working conditions that promote public health. Am J Public Health. (2016) 106:989–95. 10.2105/AJPH.2016.303138 27077343 PMC4880255

[80] Schneider SK. The policy role of state professional licensing agencies: perceptions of board members. Public Administration Quarterly. (1986) 9:414–33.

[81] Hampton T. Experts address risk of physician suicide. JAMA. (2005) 294:1189–91. 10.1001/jama.294.10.1189 16160124

[82] Dewa CS Loong D Bonato S Thanh NX Jacobs P. How does burnout affect physician productivity? a systematic literature review. BMC Health Serv Res. (2014) 14:325. 10.1186/1472-6963-14-325 25066375 PMC4119057

[83] WilliamsESKonradTRLinzerMMcMurrayJPathmanDEGerrityM. Physician, practice, and patient characteristics related to primary care physician physical and mental health: results from the physician worklife study. Health Serv Res. (2002) 37:119–41. 10.1111/1475-6773.0000711949917

[84] Hartzband P Groopman J. Physician burnout, interrupted. N Engl J Med. (2020) 382:2485–7. 10.1056/NEJMp2003149 32356624

